# Single molecule analysis indicates stimulation of MUTYH by UV-DDB through enzyme turnover

**DOI:** 10.1093/nar/gkab591

**Published:** 2021-07-07

**Authors:** Sunbok Jang, Matthew A Schaich, Cindy Khuu, Brittani L Schnable, Chandrima Majumdar, Simon C Watkins, Sheila S David, Bennett Van Houten

**Affiliations:** UPMC Hillman Cancer Center, University of Pittsburgh, Pittsburgh, PA 15213, USA; Department of Pharmacology and Chemical Biology, School of Medicine, University of Pittsburgh, Pittsburgh, PA 15213, USA; UPMC Hillman Cancer Center, University of Pittsburgh, Pittsburgh, PA 15213, USA; Department of Pharmacology and Chemical Biology, School of Medicine, University of Pittsburgh, Pittsburgh, PA 15213, USA; Department of Chemistry and Biochemistry, Molecular, Cell and Development Graduate Group, University of California, Davis, One Shields Avenue, Davis, CA 95616, USA; UPMC Hillman Cancer Center, University of Pittsburgh, Pittsburgh, PA 15213, USA; Molecular Biophysics and Structural Biology Graduate Program, University of Pittsburg, PA 15260, USA; Department of Chemistry and Biochemistry, Molecular, Cell and Development Graduate Group, University of California, Davis, One Shields Avenue, Davis, CA 95616, USA; UPMC Hillman Cancer Center, University of Pittsburgh, Pittsburgh, PA 15213, USA; Center for Biologic Imaging, University of Pittsburgh, Pittsburgh, PA 15261, USA; Department of Chemistry and Biochemistry, Molecular, Cell and Development Graduate Group, University of California, Davis, One Shields Avenue, Davis, CA 95616, USA; UPMC Hillman Cancer Center, University of Pittsburgh, Pittsburgh, PA 15213, USA; Department of Pharmacology and Chemical Biology, School of Medicine, University of Pittsburgh, Pittsburgh, PA 15213, USA; Molecular Biophysics and Structural Biology Graduate Program, University of Pittsburg, PA 15260, USA

## Abstract

The oxidative base damage, 8-oxo-7,8-dihydroguanine (8-oxoG) is a highly mutagenic lesion because replicative DNA polymerases insert adenine (A) opposite 8-oxoG. In mammalian cells, the removal of A incorporated across from 8-oxoG is mediated by the glycosylase MUTYH during base excision repair (BER). After A excision, MUTYH binds avidly to the abasic site and is thus product inhibited. We have previously reported that UV-DDB plays a non-canonical role in BER during the removal of 8-oxoG by 8-oxoG glycosylase, OGG1 and presented preliminary data that UV-DDB can also increase MUTYH activity. In this present study we examine the mechanism of how UV-DDB stimulates MUTYH. Bulk kinetic assays show that UV-DDB can stimulate the turnover rate of MUTYH excision of A across from 8-oxoG by 4–5-fold. Electrophoretic mobility shift assays and atomic force microscopy suggest transient complex formation between MUTYH and UV-DDB, which displaces MUTYH from abasic sites. Using single molecule fluorescence analysis of MUTYH bound to abasic sites, we show that UV-DDB interacts directly with MUTYH and increases the mobility and dissociation rate of MUTYH. UV-DDB decreases MUTYH half-life on abasic sites in DNA from 8800 to 590 seconds. Together these data suggest that UV-DDB facilitates productive turnover of MUTYH at abasic sites during 8-oxoG:A repair.

## INTRODUCTION

The DNA glycosylase MUTYH plays a critical role in genome maintenance by preventing mutations associated with 8-oxo-7,8-dihydroguanine (8-oxoG). MUTYH is unusual among base excision repair (BER) glycosylases due to its specific activity for removal of an *undamaged* nucleobase: an adenine (A) misincorporated opposite 8-oxoG (1). MUTYH-mediated base excision initiates the long-patch (LP) BER pathway, followed by the coordinated action of several proteins, including APE1, PCNA and DNA polymerase lambda (Pol λ), as well as the scaffolding function of X-Ray Repair Cross Complementing protein 1 (XRCC1) (2–5). Additionally, high mobility group box 1 (HMG1) further helps direct the pathway away from short-patch BER and towards long-path BER (6). The combined BER activity restores a proper 8-oxoG:C substrate for 8-oxoG glycosylase (OGG1) that excises 8-oxoG providing the opportunity for recreation of the originally coded G:C base pair. MUTYH binding of 8-oxoG:A mispair substrate and 8-oxoG:AP (abasic site) product prevents inappropriate and promutagenic OGG1 catalyzed removal of 8-oxoG from these contexts (7–9). The importance of MUTYH in genome maintenance is underscored by the correlation between inheritance of functionally compromised variants and colorectal cancer, referred to as *MUTYH*-associated polyposis (MAP) (10).

The structural similarity between 8-oxoG*_syn_*:A*_anti_* mispairs and undamaged thymine (T):A bps within DNA makes the lesion location and repair process of MUTYH particularly remarkable (11–13). Adenine excision is known to occur within the N-terminal catalytic domain of MUTYH, while 8-oxoG recognition resides primarily in a C-terminal 8-oxoG recognition domain (14–16). In a recent study, using single molecule microscopy, *in vitro* glycosylase and cellular repair assays, the Lee and David laboratories showed that *Escherichia coli* MutY detects 8-oxoG:A mispairs via the unique major groove positioning of the 2-amino group 8-oxoG using a conserved histidine residue within an ‘FSH’ loop of the C-terminal 8-oxoG recognition domain (13). Notably, identification of this distinct chemical signal of 8-oxoG:A mismatches would be anticipated to be further complicated in eukaryotic genomes, where the damaged mismatch can be sequestered within chromatin.

Prior work by Matsumoto *et al.* has shown that the nucleotide excision repair (NER) protein, UV-DDB, that binds to UVC-induced pyrimidine photoproducts, can access lesions hidden within nucleosomes by shifting the register of the damaged DNA strand and exposing the lesion for excision (17). Surprisingly, in addition to exhibiting high affinity for cyclobutane pyrimidine dimers and pyrimidine-pyrimidone 6–4 photoproducts, UV-DDB exhibits high specificity for abasic sites (18–20) suggesting a broader role in repair. Indeed, several studies have suggested a critical role for UV-DDB in maintaining genomic integrity (21,22). For instance, loss of UV-DDB has been associated with the incidence of spontaneous tumors in mice, and in humans, a low expression level is correlated with poor cancer prognosis (22–24).

We have previously demonstrated that UV-DDB also plays an unexpected role in BER by stimulating the enzymatic turnover of OGG1 *in vitro* (25). Through single molecule visualization of OGG1 on damage containing DNA tightropes and imaging in cells, we showed that UV-DDB colocalizes with OGG1 on abasic sites. Biochemical assays revealed that UV-DDB facilitates the turnover of OGG1 and APE1 from damaged DNA and also stimulated the activity of Pol ß. This study also provided preliminary data suggesting UV-DDB can stimulate MUTYH. Cellular experiments using a novel method to introduce 8-oxoG at telomeres (26) indicated that UV-DDB arrives at sites of 8-oxoG prior to OGG1 (25). Taken together, these data led us to propose a base damage sensing role for UV-DDB (25). Moreover, the ability of UV-DDB to expose occluded lesions (17), and to have specificity for 8-oxoG:C and 8-oxoG:A sites and to stimulate BER, suggests that this protein may act as a ‘first-responder’ to mediate repair of oxidative damage in the genome. These results prompted us to further investigate the role of UV-DDB in the repair of 8-oxoG:A mismatches mediated by MUTYH. It is known that MUTYH exhibits slow substrate turnover due to its exceptionally high affinity for its 8-oxoG:AP site product (27). In this work, we present mechanistic data showing UV-DDB stimulates MUTYH turnover with 8-oxoG:A substrates. Using single molecule analyses and *in vitro* biochemical assays, we revealed that UV-DDB interacts with MUTYH similarly to OGG1 and promotes dissociation of MUTYH from AP site product to enhance overall BER of 8-oxoG:A mismatches.

## MATERIALS AND METHODS

### Expression and purification of recombinant UV-DDB and MUTYH

Recombinant full-length UV-DDB (DDB1–DDB2 heterodimer) was expressed in Sf9 cells coinfected with recombinant baculovirus of His_6_-DDB1 and DDB2-Flag, as performed previously (28). Briefly, His_6_-DDB1 and DDB2-Flag were purified using a 5 mL His-Trap HP column pre-charged with Ni^2+^ (GE Healthcare) and anti-FLAG M2 affinity gel (Sigma). The pooled anti-FLAG eluates were size fractionated on a HiLoad 16/60 Superdex 200 column (Amersham Pharmacia) in UV-DDB storage buffer (50 mM HEPES, pH 7.5, 200 mM KCl, 1 mM EDTA, 0.5 mM PMSF, 2 mM DTT, 10% glycerol and 0.02% sodium azide). Purified fractions of DDB1–DDB2 complex from the Superdex200 were aliquoted and flash frozen with liquid nitrogen and stored at −80°C.

Two versions of mouse MUTYH were purified: a hexa-histidine tag intact for single molecule experiments and another where the tag was cleaved using thrombin for *in vitro* kinetics experiments. Hexa-histidine tagged MUTYH was overexpressed in BL21(DE3) co-expressing the *pRKISC* iron-sulfur cluster assembly plasmid using the protocol reported previously (29). Briefly, the cell pellets harvested from 4 L of LB growth media were resuspended in 35 mL of resuspension buffer (20 mM Tris buffer pH 7.5, 10% glycerol) and lysed by sonication. After centrifugation, solid NaCl and imidazole were added to final concentrations of 1 M and 20 mM, respectively. The supernatant was filtered and loaded onto an AKTA FPLC equipped with three 1 mL HisTrap FF columns (GE Healthcare) connected in series, pre-equilibrated in Ni(II) binding buffer (20 mM sodium phosphate buffer pH 7.4, 0.5 M NaCl, 20 mM imidazole). The protein was eluted over 5 column volumes over a linear gradient of 0–100% Ni (II) elution buffer (20 mM sodium phosphate buffer pH 7.4, 0.5 M NaCl, 500 mM imidazole). Peak fractions were pooled and buffer exchanged into Heparin Buffer A (20 mM Tris–HCl pH 7.5, 10% glycerol) and further purification was carried out using a 1ml HiTrap Heparin column (GE Healthcare) on an AKTA FPLC using a linear gradient of 0% - 100% Heparin Buffer B (20 mM Tris pH 7.5, 10% glycerol, 1 M NaCl). Fractions were analyzed for protein concentration and [4Fe4S] cluster loading by measuring absorbances at 280 nm and 410 nm respectively. Fractions with a cluster loading of >70% were pooled and concentrated to ∼20 μM. For cleavage of the hexa-histidine tag, MUTYH was treated with 2 U thrombin/mg of protein and cleaved protein was purified away from tagged protein using a reverse Ni(II) column prior to the Heparin column. An equal volume of pre-chilled 50% glycerol was added to the purified protein sample to yield a final buffer concentration of 10 mM Tris pH 7.5, 25% glycerol and 225 mM NaCl. Purified MUTYH was aliquoted into single use aliquots and flash frozen using liquid nitrogen for storage at –80ºC. Activity of each MUTYH purification was measured via active site titration and varied between 33% and 50% (29). These activities were used to quantify MUTYH concentration used in all assays.

APE1 was purchased from abcam (Cambridge, United Kingdom).

All experiments were performed with three different preparations of UV-DDB and three different preparations of MUTYH and gave similar results. All biochemical studies used untagged MUTYH and single molecule studies used the His-tagged version.

### DNA substrate preparation

#### 37 bp DNA duplexes for excision assay and EMSA

The following oligonucleotides sequences (Z = 8-oxoG and X = tetrahydrofuran) were used:

8-oxoG37-top: 5′-CCG AGT CAT TCC TGC AGC GA**Z** TCC ATG GGA GTC AAA T-3′

A37-bottom: 5′-A TTT GAC TCC CAT GGA **A**TC GCT GCA GGA ATG ACT CGG-3′-6FAM

THF37-bottom: 5′-A TTT GAC TCC CAT GGA **X**TC GCT GCA GGA ATG ACT CGG-3′-6FAM

THF37-top: 5′-CCG AGT CAT TCC TGC AGC G**X**G TCC ATG GGA GTC AAA T-3′

#### 37 bp oligonucleotide for creating an 8-oxoG:A site in AFM studies

5′-CCG AGT CAT TCC TGC AGC GAG **Z**CC ATG GGA GTC AAA T-3′ (**Z**= 8-oxoG)

8-oxoG37(G:A) was prepared by annealing 8-oxoG37-top (purchased from Trilink, USA) and A37-bottom (purchased from IDT, USA). THF8-oxoG37 was prepared by annealing 8-oxoG37-top and THF37-bottom (purchased from IDT, USA). Annealing reactions were done at 95°C for 5 min in 10 mM Tris–HCl, pH 8.0, and 100 mM KCl and then cooled to room temperature slowly for 4 h by turning off the heating device.

#### Plasmids containing site-specific lesions

Plasmids containing single site-specific THF adduct or 8-oxoG:A were prepared as described previously (30,31). Briefly, purified pSCW01 plasmids were nicked by *Nt.BstNBI* to create a 37-base gap. A 37mer containing a single abasic site (THF37-top, above) was annealed into this gap and the backbone was sealed with T4 DNA ligase. The THF arrays were prepared using the defined lesion plasmid described above. Lesion-containing pSCW01 was linearized via restriction digest by *XhoI* (NEB) then incubated with T4 DNA ligase (NEB) to achieve long (>40 kb) tandemly ligated products with one THF site every 2 kb. To generate a 538 bp DNA fragment containing an 8-oxoG:A site 30% from one end, a 37 base oligonucleotide indicated above was ligated into pSCW01 gapped plasmid. The plasmid was digested with XmnI and PciI restriction enzymes (NEB) and the fragment was purified as described previously (31).

### MUTYH excision assay

Reactions were carried out in a volume of 10 μL containing MUTYH excision buffer (20 mM HEPES, pH 7.9, 50 mM NaCl, 1 mM MgCl_2_, 1 mM DTT), 50 nM of fluorescein-labeled 8-oxoG37(G:A) containing duplex DNA and the indicated amount of MUTYH and UV-DDB. Reactions were incubated at 37°C for each time point (up to 4 h) and rapidly quenched by adding an equal volume of gel loading buffer (2× formamide dye solution), heated at 95°C for 5 min, then cooled on ice for 5 min. 0.1 M NaOH was included in the quenching step to induce DNA nicking at the abasic site. The reaction product was separated by electrophoresis on 10% denaturing polyacrylamide gel and visualized using a laser scanner for fluorescence (Typhoon, Amersham). The substrate and product bands were quantified using ImageJ.

### Electrophoretic mobility shift assays (EMSA)

MUTYH-DNA reaction was prepared by combining 8 nM of 37 bp THF8-oxoG DNA with 20nM of MUTYH in reaction buffer (20 mM HEPES, pH 7.5, 150mM NaCl, 5 mM DTT, 0.5 mg/ml BSA, and 5% glycerol), incubating for 10 min at RT, then mixing with increasing amounts of UV-DDB (0–64 nM) in a final reaction volume of 10 μL. Each reaction was incubated for 30 min at RT then immediately loaded on two pre-run 5% polyacrylamide (37.5:1, acrylamide : bis) native gels and run at 100 V for 50 min at 4°C in 1/2× TBE (4.5 mM Tris, 44.5 mM boric acid, 1 mM EDTA, pH 8.4). DNA bands were visualized using a laser scanner for fluorescence (Typhoon, Amersham). The percentage of total DNA bound by each protein was determined by measuring the band intensity present in the bound states and dividing by the total band intensity in the lane. Background signals from blank regions of the gel were subtracted from the signal intensities obtained from bands. The percentage of DNA bound in each reaction was plotted against the concentration of UV-DDB.

### DNA tightrope assay

Single-molecule DNA tightrope assay was performed as described previously (31). Briefly, poly-l-lysine (Wako Pure Chemicals) coated silica beads (5 μm; Polysciences Inc.) were deposited onto a PEG-treated coverslip (24 × 40 mm; Corning) in a custom flow cell. Defined lesion (abasic site) substrates were strung up across the beads via hydrodynamic flow. DNA substrates were made by tandem ligation of pSCW01 plasmid (∼2 kb) with a single, site specific THF modification and proximal biotinylated thymine.

#### Protein labeling

Prior to imaging, purified His-tagged MUTYH was labelled with secondary antibody-coated 605 nm quantum dots (Qdots; Invitrogen) through α-His primary antibody (Qiagen). For UV-DDB-facilitated dissociation experiments, quantum dot-labeled MUTYH was injected into the flow cell at final concentrations of 2.6 nM with 1× UV-DDB conjugated to goat α-Flag primary antibody. For colocalization experiments, purified UV-DDB was conjugated to streptavidin coated 705 nm Qdots through biotinylated goat α-Flag primary antibody (Bethyl), and quantum dot-labelled MUTYH or UV-DDB were injected into the flow cell at final concentrations of 2.6 or 3.1 nM, respectively. We conjugated each protein in separate reaction tubes and injected into the flow cell separately for dual-color experiments; flow was stopped during the observation period. Furthermore, we performed a control experiment to check whether there is any unwanted interaction between 705Qdot-labeled UV-DDB (DDB1 is His-tagged) and α-His primary antibody conjugated to 605Qdots previously (25). 705Qdot conjugated UV-DDB was injected into the flow cell and then injected 605Qdot conjugated with α-His primary antibody, and observed for 4 h. During this time, 20 particles were recorded, but none were co-localized. All binding experiments were carried out in tightrope buffer (25 mM HEPES, pH 7.5, 150 mM NaCl, 0.1 mg/ml BSA (Roche), 50 nM biotin, and 1 mM DTT).

#### Data collection

Labeled proteins were visualized using oblique angle fluorescence (Nikon Eclipse Ti inverted microscope with Nikon 100× TIRF objective and 1.45 numerical aperture) with a 488 nm laser (power 1–2 mW) at the back focal plane to excite Qdots and the appropriate emission filter (Chroma) applied at RT. Movies were taken for 5 min with frame rates between ∼11 and 12.5 fps.

#### Data analysis

Images were acquired using Nikon Elements (4.2) and exported as TIFF stacks for kymograph processing and analysis in ImageJ (NIH). Differences between dissociation rates and motility fractions were assessed by one-way ANOVA. For half-life determination, the data were fit to a single-exponential decay function in GraphPad Prism. Errors of fits were also calculated with GraphPad Prism.

The mean squared displacement (MSD) was calculated for all motile phases using custom scripts in MATLAB:

$$\begin{equation*}MSD\ (n\Delta t) = \frac{1}{{N - n}}\ \mathop \sum \limits_{i\ = \ 1}^{N - n} {\left( {{x_{i + n}} - {x_i}} \right)^2}\end{equation*}$$

where *N* is total number of frames in the phase, *n* is the number of frames at a given time step, Δ*t* is the time increment of one frame, and *x_i_* is the particle position in the *i*th frame (32). The diffusion coefficient (*D*) was determined by fitting a linear model of one-dimensional diffusion to the MSD plots:

$$\begin{equation*}MSD\ (n\Delta t) = \ 2D\ (n\Delta t) + y\end{equation*}$$

where *y* is a constant (*y*-intercept). Fittings resulting in *R*^2^ <0.8 or using <10% of the MSD plot were not considered.

### Atomic force microscopy (AFM)

#### Sample preparation

All buffers were filtered through 0.02 μm membrane filter (Whatman). UV-DDB-MUTYH-DNA reactions consisted of 140 nM of 538 bp substrate, 350 nM UV-DDB, and 350 nM MUTYH, for a final volume of 10 μl in MUTYH AFM buffer (20 mM HEPES, pH 7.9, 50 mM NaCl, 1 mM MgCl_2_ and 1.4 mM DTT). MUTYH was added first and incubated at room temperature for 10 min. UV-DDB was then added, and the reaction incubated at room temperature for 15 min. The binding reaction was diluted 1:25 in AFM Deposition buffer (25 mM HEPES, pH 7.5, 25 mM NaOAc, 10 mM Mg(OAc)_2_) that was heated at 65°C and cooled to room temperature. Free protein was diluted to 40 nM in AFM deposition buffer. The dilution was pipetted onto freshly cleaved mica, rocked gently for 30 s, washed with 1mL of filtered H_2_O, and dried under a gentle stream of nitrogen.

#### Generation of the standard curve

Free protein was diluted to 40 nM in AFM deposition buffer. A total of between 600 and 1000 particles were analyzed for each protein. Gaussian was fit to determine the average volume for each protein. UvrA (purified as published (33,34)), UvrB (purified as published (35)), and OGG1 (purchased from Novus Biologicals) were used to generate the standard curve.

#### Data collection

Data was collected using ScanAsyst PeakForce Tapping mode in air on a Multimode V Microscope with an E scanner (Bruker). 1 × 1 μm images were taken with pixel size 512 × 512 using SCANASYST-AIR probes with 2 nm radius tip (Bruker).

#### Volume analysis

The volumes of the free protein and the protein–DNA complexes were determined using Image SXM. For free proteins, the volume was calculated:

$$\begin{equation*}Volume\ = \left( {< H >- \ B} \right)\, x\ A\end{equation*}$$

where *<H>* is the average height of the particle, *B* is the background of the overall image, and *A* is the area of the particle determined by thresholding above the background.

For protein–DNA complexes, the volume of the DNA was estimated and subtracted from the total volume of the complex. Image SXM was used to outline the complex and select unbound regions of DNA equal to the length of the complex (on either side of the complex). The volume of the protein was thus calculated:

$$\begin{equation*}{\rm{\ }}{V_{protein}} = {V_{complex}}\ - \frac{{{V_{DNA1}} + {V_{DNA2}}}}{2}\end{equation*}$$

The linear regression from the standard curve was used to determine the molecular weight of each complex. All molecular weights were plotted and fit to a Gaussian.

## RESULTS AND DISCUSSION

### UV-DDB discriminates a wide range of DNA lesions from non-damaged DNA

Previous work on UV-DDB by several groups (18–20) including our own (22,25) showed that UV-DDB has high affinity for abasic sites, in addition to UV-induced photoproducts. While previous work indicated that UV-DDB had no detectable affinity for 8-oxoG:C or 8-oxoG:A pairs (19), we have found that the addition of physiological concentrations of Mg^2+^, while decreasing overall binding of UV-DDB to DNA, greatly enhances specificity for these two lesions by facilitating DNA sliding resulting in decreased affinity for non-damaged DNA (22,25).

### UV-DDB stimulates MUTYH excision activity

**Figure 1.**
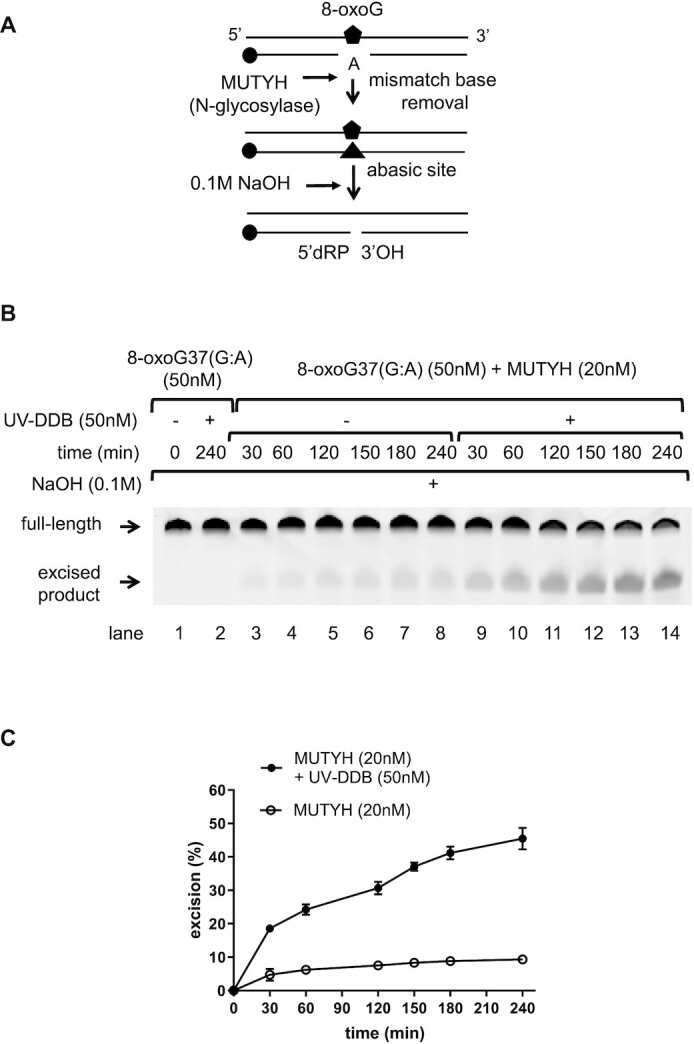
UV-DDB stimulates MUTYH by binding specifically to 8-oxoG:A. (**A**) Schematic representation of the DNA substrate containing 8oxoG:A and the proposed reaction scheme. Circle represents a 3′-FAM moiety (**B**) Stimulation of MUTYH excision kinetics by UV-DDB. MUTYH (20 nM) was incubated with dsDNA (50nM) containing 8-oxoG:A in the absence (−) or presence (+) of UV-DDB (50nM) at 37°C. Aliquots were withdrawn at each time point and analyzed on a 10% denaturing polyacrylamide gel. Positions of the un-cleaved full-length substrate and excised product are indicated by arrows. (**C**) Quantification of the stimulation of MUTYH excision kinetics by UV-DDB in (B). Excision product formation was quantified using ImageJ software. The excision percentage was plotted as mean ± SD from three independent experiments, each run on duplicate gels.

In a previous study, we showed that UV-DDB stimulates OGG1 removal of 8-oxoG by increasing the turnover of OGG1 (25). In this same study we also discovered that UV-DDB stimulated the excision activity of MUTYH for A across from 8-oxoG. In this present study we wanted to further examine the mechanism of MUTYH stimulation by UV-DDB. To determine the effect of UV-DDB on MUTYH-catalyzed A base excision paired with 8-oxoG, a limiting amount of MUTYH (20nM) was incubated with excess 8-oxoG37(G:A) duplex oligonucleotide (50 nM) in the absence or the presence of UV-DDB up to 4 h (Figure 1, see also Supplementary Figure S1). In this assay, the MUTYH-processed DNA was treated with NaOH to convert AP sites to nicks before running the samples on denaturing PAGE. MUTYH showed burst kinetics consistent with previous studies (36), but extended time course showed little if any turnover. When UV-DDB (50 nM) was added to MUTYH, A excision increased ∼ 4–5-fold (Figure 1B and C). Purified UV-DDB by itself showed no detectable DNA glycosylase activity on 8-oxoG37(G:A) (lane 2 at Figure 1B). These excision kinetic data suggest that UV-DDB may stimulate the turnover of MUTYH activity through facilitated product release during MUTYH catalytic cycle during BER. These kinetic results alone, however, cannot fully describe the mechanism for how this stimulation occurs. For instance, presence of UV-DDB may also increase the stability of MUTYH at longer timepoints via a protein crowding mechanism (37). Therefore, MUTYH stimulation was investigated in more detail using electrophoresis gel mobility shift assays (EMSA) and single molecule studies described below.

### UV-DDB facilitates dissociation of MUTYH from abasic sites in DNA

**Figure 2.**
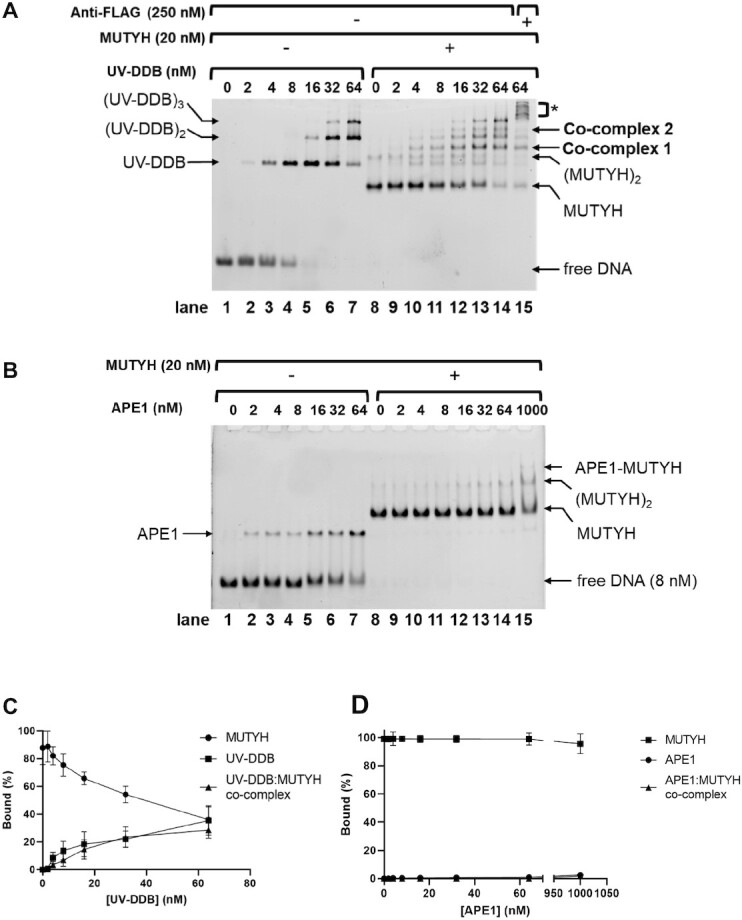
UV-DDB facilitates MUTYH dissociation via co-complex formation. (**A**) UV-DDB forms co-complexes with MUTYH and displaces MUTYH on abasic sites, shown by EMSA. Binding reactions of THF8oxoG37 and increasing amounts of UV-DDB with or without MUTYH were separated by native PAGE. Protein-DNA complexes were identified based on band migration and labelled accordingly. In lane 15, addition of 250 nM anti-FLAG antibody caused a supershift in UV-DDB containing species, marked with an asterisk (see Supplementary Figure S3). Representative gel shown from three independent experiments. (**B**) Binding reactions of THF8oxoG37 of APE1 with and without MUTYH were assayed by native PAGE. Even at up to 1 μM APE1, minimal MUTYH dissociation was observed. After running on a sequencing gel (Supplementary Figure S4) MUTYH at 20 nM prevented nearly all APE1 endonuclease activity (**C**) Quantification of (A) from lane 8 to 14. Percent bound of total DNA by all UV-DDB species (including monomer, dimer, and trimer), MUTYH species (including monomer and dimer), and co-complex (including co-complex 1 and co-complex 2) are plotted as a function of UV-DDB concentration. Data shown as the mean of two measurements from three experiments ± SD. (**D**) Quantification of (C) from lane 8–15. Data shown as the mean of two measurements from three experiments ± s.d.

Our previous study has suggested that UV-DDB facilitates the dissociation of OGG1 from THF-containing substrates and APE1 from substrates with incised THF, thus increasing product release rates which may contribute to increasing their rates of turnover (25). We sought to investigate this hypothesis using an EMSA. MUTYH (20 nM) was bound to a THF:8-oxoG-containing duplex DNA substrate (8 nM) by incubating these two components for 10 min at RT. This AP site analogue (THF) was chosen because it forms a stable complex with MUTYH. Upon incubation of MUTYH with the oligonucleotide duplex, >80% of the DNA was in the form of a complex with MUTYH (Figure 2, lane 8). UV-DDB (0–64 nM) was added in the absence of MUTYH (lane 1–7) or presence of MUTYH (lane 8–14). After 20 additional minutes of incubation at RT, UV-DDB formed co-complexes with MUTYH and, at higher concentrations, facilitated the dissociation of the DNA substrate from MUTYH (Figure 2A and C, lanes 12–14). To confirm the identities of these bands containing both UV-DDB and MUTYH, we incubated the highest concentration with 250 nM anti-FLAG antibody, causing a supershift for bands containing UV-DDB (Figure 2A, lane 15 and Supplementary Figure S3). To compare the mechanism of how UV-DDB facilitates product dissociation of MUTYH to the way that APE1 facilitates the handoff, similar concentrations of APE1 were also used, up to 1000 nM (Figure 2B and D, lanes 1–15). Even at the highest concentration of APE1, MUTYH barely dissociated from its product. In fact, 20 nM MUTYH protected the THF site from cleavage by APE1 compared to the lanes containing APE1 alone, which even at 2 nM significantly cleaved the THF-containing substrate (Supplementary Figure S4).

The high affinity of MUTYH for AP sites makes it relatively resistant to displacement by APE1 (30) compared to OGG1, which is readily displaced by APE1 (31). In fact, despite demonstrations of a direct MUTYH-APE1 interaction (32), 100-fold excess APE1 was needed to mediate displacement (33). We also found that while 20–30-fold excess of APE1 over MUTYH was necessary for MUTYH turnover, UV-DDB at amounts of 0.5–2.5 fold excess were sufficient to allow multiple cycles of MUTYH catalysis (Figure 1 and Supplementary Figure S2). In addition, even under optimal conditions with APE1, UV-DDB could stimulate MUTYH turnover (Supplementary Figure S2). These EMSA analyses confirmed that equal molar concentrations of UVDDB as compared to MUTYH was capable of displacing MUTYH from abasic sites (lane 12, Figure 2A). Because the enzymes exhibit disparate *K*_d_ values for this substrate (4 nM for UV-DDB compared to 0.05 nM for MUTYH), differences in *K*_d_ values alone cannot fully account for how this handoff occurs at equimolar concentrations. Instead, we hypothesize that UV-DDB interacts with OGG1 (25) and MUTYH glycosylases bound to their products, facilitating their dissociation. Thus, UV-DDB may stimulate the turnover of other glycosylases with a similar facilitated diffusion mechanism, perhaps analogous to UV-DDB’s known ability to shift the registry of damaged DNA tightly bound to nucleosomes to make the DNA damage more accessible for repair (17).

### Single molecule analysis of MUTYH dissociation by UV-DDB

**Figure 3.**
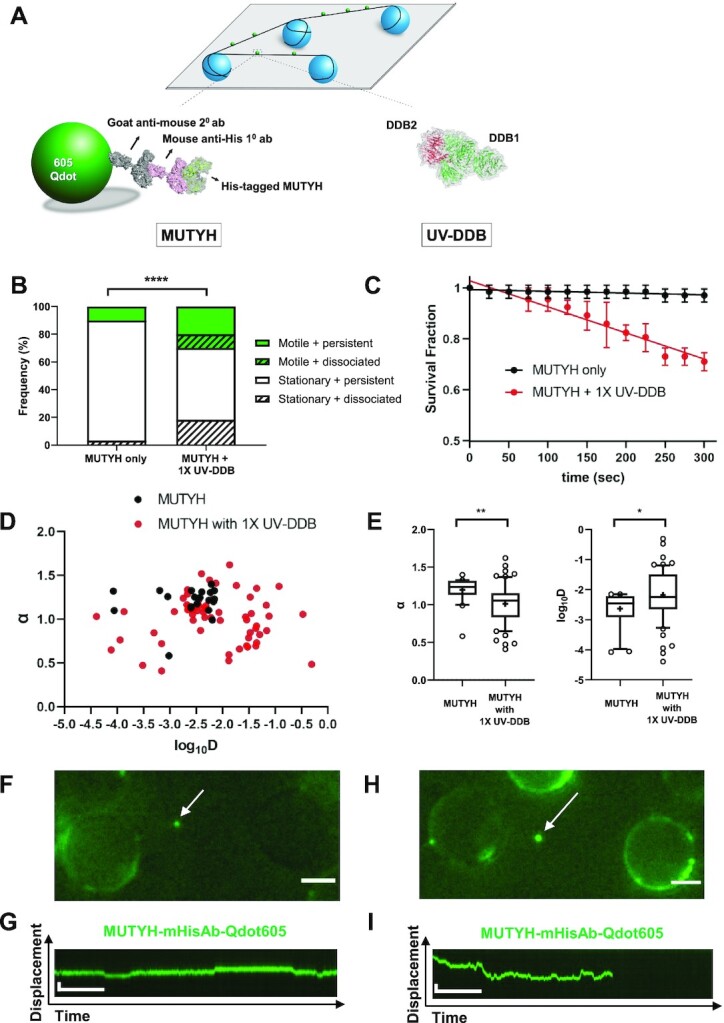
Single molecule analysis reveals that UV-DDB stimulates turnover of MUTYH by facilitated dissociation. (**A**) Experimental design of DNA tightrope assay to study UV-DDB induced mobility and dissociation of MUTYH (top). Long DNA substrates with defined abasic sites (THF) every 2 kb are suspended between silica beads (bottom, left). His-tagged MUTYH is labelled with primary mouse-anti-His antibody and secondary goat-anti-mouse antibody conjugated to a 605nm Qdot. (bottom, right) un-labeled UV-DDB. (**B**) Stack bar graph showing the fraction of motile (green) versus stationary (white) and persistent (solid) vs. dissociating (diagonal lines) particles of 605Qdot labelled MUTYH in the absence (−) or presence (+) of un-labeled 1× UV-DDB on abasic (THF) DNA during 300s observation. (****P< 0.0001 by χ2 test). (**C**) Effects of UV-DDB on the lifetimes of MUTYH-DNA complexes. Data plotted as the mean ± SEM from three independent experiments. For each condition, survival fraction decay is fit to a single exponential decay function to obtain the half-life and errors shown are the errors of the fit. (**D**) Anomalous diffusion exponent (α) versus diffusion coefficient (log_10_*D*) plotted for MUTYH (black filled circles) and MUTYH with 1× UV-DDB (red filled circles). (**E**) Box and whisker plot (10–90 percentile) of left, the Anomalous diffusion exponent (α) and right, the diffusion coefficient (log_10_*D*) calculated for MUTYH only (*n* = 20 phases) and MUTYH with 1× UV-DDB (*n* = 61 phases) phases on long DNA substrates with defined abasic sites (THF) every 2 kb. +, sample mean, *P < 0.1, **P < 0.01 by two-tailed Student's *t* test. (**F**) Image of 605Qdot-labled MUTYH (green) on abasic (THF) tightrope suspended between beads in the 1× presence of unlabeled UV-DDB. Scale bar represents 2.5 μm. Arrow points to motile MUTYH particle. See corresponding video 1. (**G**) Kymograph of 605Qdot-labeled MUTYH (green) with motile particle (F). Horizontal and vertical scale bars represent 50 s and 2 kb, respectively. (**H**) Image of 605Qdot-labled MUTYH (green) on abasic (THF) tightrope suspended between beads in the 1× presence of un-labelled UV-DDB. See corresponding Video 2. Scale bar represents 2.5 μm. Arrow points to motile/ dissociated MUTYH particle. (**I**) Kymograph of 605Qdot-labeled MUTYH (green) with motile and dissociated particle (H). Horizontal and vertical scale bars represent 50s and 2kb, respectively.

In order to further test the hypothesis that UV-DDB may increase the rate of MUTYH dissociation from abasic (THF) sites, we turned to single molecule analysis. In these experiments, DNA tightropes containing abasic sites were suspended from 5 micron beads and the rates of dissociation of quantum dots (Qdots)-labelled MUTYH was monitored in real-time. MUTYH was labeled with 605 nm Qdots using an antibody sandwich approach (38) in which a primary mouse anti-His antibody was bound to a His-tagged MUTYH protein, then goat anti-mouse secondary antibody-coated 605 nm Qdots (Figure 3A). Qdot-labeled MUTYH was observed in the absence or presence of unlabeled UV-DDB on DNA tightropes containing one abasic (THF) site every 2 kb (Materials and Methods) for a total of 5 min, and the number of Qdot-labeled proteins dissociating during that time was noted. MUTYH displayed three-fold increase in the frequency of dissociation (during 5-min observation window) when same amount of UV-DDB was added (Figure 3B). Addition of equimolar of UV-DDB to MUTYH decreased the half-life of MUTYH from 8900 ± 4700 s to 590 ± 40 s (Figure 3C). The relatively large error for the former is due to a very slow off rate which when fitted to an exponential decay gave a large uncertainty.

We found that in addition to increasing the rate of dissociation of MUTYH from abasic sites, UV-DDB also increased the motility of MUTYH on DNA. Compared to MUTYH alone (10% motile) addition of UV-DDB increased MUTYH motility ∼3-fold. The diffusive behavior of each motile MUTYH molecule was further analyzed by characterization of its anomalous diffusion exponent (α factor) and diffusion coefficient (D) in (Figures 3D and E). The mean α factor of MUTYH only and MUTYH + 1× UV-DDB is 1.19 and 1.01, and its mean diffusion coefficient is 3.75 × 10^–3^ μm^2^/s and 3.01 × 10^–2^ μm^2^/s, respectively. Examples of UV-DDB induced motility and motile dissociation of MUTYH are shown in Figure 3F and G, and H amd I, respectively. Also see corresponding Videos 1 and 2. These data when combined with the EMSA results indicate that UV-DDB can readily dissociate MUTYH directly from abasic sites and/or by increasing its motility on DNA.

### Single molecule analysis of colocalization of UV-DDB with MUTYH on DNA containing abasic sites

**Figure 4.**
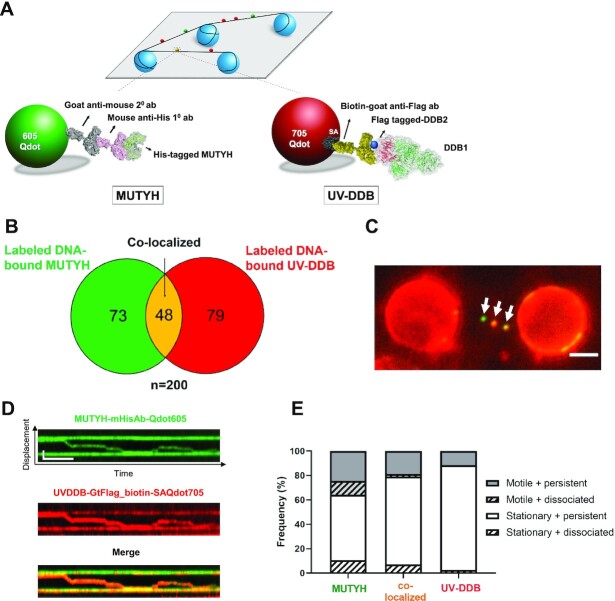
Single molecule co-localization of UV-DDB with MUTYH on abasic DNA tightropes. (**A**) Schematic of the DNA tightrope assay. Long DNA substrates with abasic sites every 2 kb were suspended between 5 μm poly-l-lysine coated silica beads. Anti-His primary antibody was used to link the His-tagged MUTYH to the 605Qdot. Biotin conjugated anti-Flag primary antibody was used to link Flag-tagged UV-DDB to streptavidin-coated 705Qdot. Uniquely labelled MUTYH and UV-DDB were observed interacting on abasic DNA tightropes in real time and their behavior and frequency of co-localization was recorded. (**B**) Venn diagram showing number of proteins that co-localized (yellow) on abasic (THF) tightropes or were observed separately for 605Qdot-labeled MUTYH (green) with 705Qdot-labeled UV-DDB (red) in the dual-color assay. (**C**) Image of co-localized (yellow) Qdot-labelled MUTYH (green) and UV-DDB (red) on abasic (THF) tightrope suspended between beads. Scale bar represents 2.5 μm. Arrow points to co-localized particle. See Video 3. (**D**) Kymograph of co-localized MUTYH and UV-DDB. Top, MUTYH (green); middle, UV-DDB (red); bottom, merged (yellow). Horizontal and vertical scale bars represent 50s and 2 kb, respectively. (**E**) Stacked bar graph showing the fraction of motile (gray) versus stationary (white) and persistent (solid) versus dissociating (diagonal lines). Results obtained with individual and co-localized particles. (MUTYH; MUTYH behavior in the presence of UV-DDB, co-local; co-localized MUTYH/UV-DDB behavior, UV-DDB; UV-DDB behavior in the presence of MUTYH). Data re-plotted as a sub-set of (B).

While the EMSA experiments suggested transient complex formation between UV-DDB with MUTYH on abasic site containing DNA, an additional series of experiments was performed to attempt to demonstrate a direct interaction between MUTYH and UV-DDB. We found no evidence of a direct interaction of UV-DDB and MUTYH by size exclusion chromatography (Supplementary Figure S5). However, since both MUTYH and UV-DDB are capable of binding to abasic sites individually, we sought to further investigate their potential transient interactions on the DNA containing abasic sites using the DNA tightrope assay at single molecule level. To this end, we used an orthogonal labelling strategy for direct dual-color fluorescence imaging of His-tagged MUTYH, labelled with 605 nm Qdots, and Flag-tagged UV-DDB, labelled with a biotinylated goat anti-Flag primary antibody and streptavidin-coated 705 nm Qdots, as previously described (25) (Figure 4A). Control experiments indicated that there is no exchange of Qdots between 605Qdot conjugated with α-His primary antibody and UV-DDB (DDB1 is His-tagged) (25).

Qdot-labeled MUTYH was first injected into a flow cell containing DNA tightropes with one abasic site (THF) every 2 kb, and Qdot-labeled UV-DDB was injected 5 min later. After injection, all flow was stopped. Owing to the transient nature (mobility and dissociation) of MUTYH and UV-DDB binding to damaged DNA, we reasoned that colocalization might be a relatively rare event. We found that colocalization within the precision of our Qdot approach of MUTYH and UV-DDB accounted for 24% of all particles (Figure 4B). Some of these colocalized molecules (Figure 4C and D) were found to diffuse on the DNA together, suggesting direct interactions on DNA; however, the overall motion and dissociation of the repair proteins did not change substantially when complexed together (Supplementary Figures S6A and B). The kymographs of merged channels (Figure 4D) showed colocalization of green and red signals, indicating transient interactions of MUTYH with UV-DDB. Additional kymographs are shown in Supplementary Figures. S7A–H and Supplementary Video 3). Also note that UV-DDB helps to induce mobility and increase dissociation of MUTYH.

**Figure 5.**
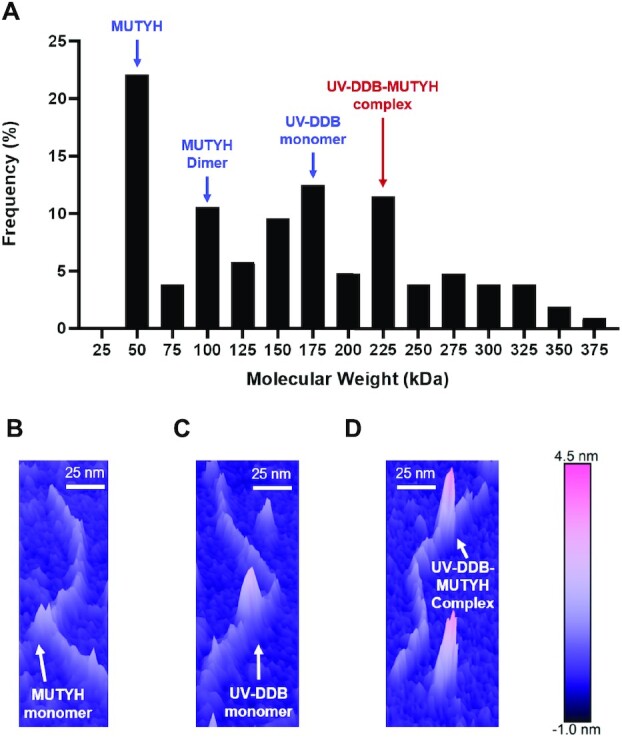
MUTYH and UV-DDB colocalize on DNA containing 8-oxoG:A damage. (**A**) Histogram showing distribution of AFM volumes of MUTYH and UV-DDB bound to DNA containing 8-oxoG:A *(n* = 104). Molecular weights corresponding to MUTYH monomer, MUTYH dimer, UV-DDB monomer, (blue) and UV-DDB-MUTYH complex (red). (**B**) Representative 3D AFM image of MUTYH bound to 8-oxoG:A DNA. (**C**) Representative 3D AFM image of UV-DDB bound to 8-oxoG:A DNA. (**D**) Representative 3D AFM image of MUTYH-UV-DDB bound to 8-oxoG:A DNA.

Atomic force microscopy (AFM) was used to confirm that UV-DDB and MUTYH co-localize at sites of 8-oxoG:A. AFM measures volumes of globular proteins, which are directly proportional to their molecular weight. In this approach, 140 nM 538 bp DNA duplex containing a 8oxoG:A site 30% from one end was mixed with 350 nM MUTYH and 350 nM UV-DDB. This solution was then diluted 1:25 and deposited on mica. Based on the measured volumes complexes consisting of MUTYH, MUTYH dimer, UV-DDB and UV-DDB-MUTYH complexes were observed (Figure 5). The standard curve for volume versus molecule weight as well as the behavior of the proteins without DNA are shown in Supplementary Figures S8 and S9. Taken together, these data suggest that UV-DDB can associate and migrate together with MUTYH on DNA.

### Working model of UV-DDB facilitated repair of 8-oxoG:A by MUTYH

**Figure 6.**
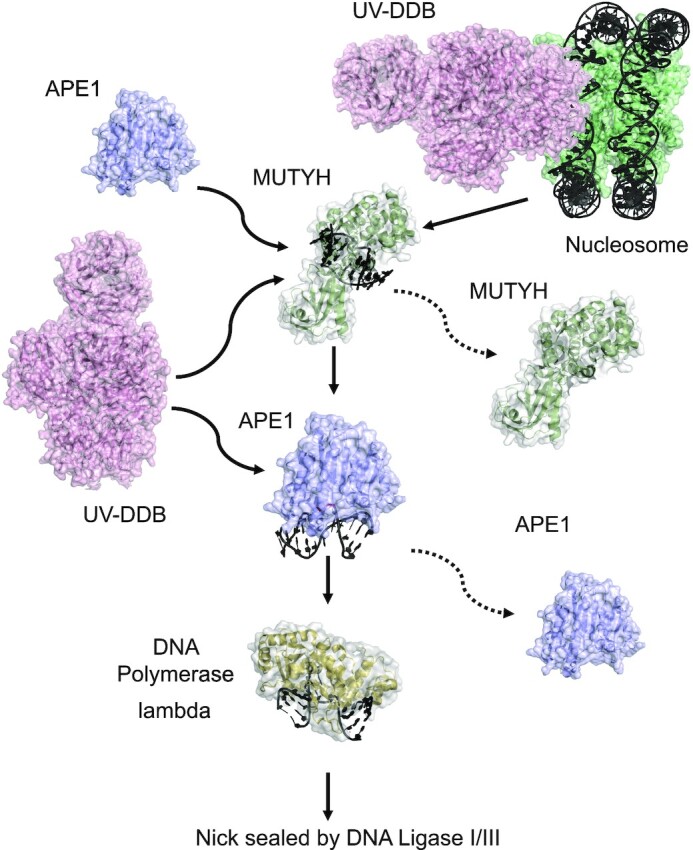
Proposed working model of UV-DDB stimulation of MUTYH in the repair of 8-oxoG. (**A**) Schematic representation of the proposed BER pathway including UV-DDB is illustrated. UV-DDB is believed to be rapidly recruited to damaged sites in chromatin and help facilitate processing by MUTYH. Biochemical and single molecule data suggest that UV-DDB transiently associates with MUTYH at 8-oxoG:abasic sites to increase its release and turnover. APE1 is expected to be necessary to incise the DNA and has been shown previously to be stimulated by UV-DDB (25). DNA pol λ then undergoes long patch repair (3), and FEN1 (not shown) processes the flap leaving a nick, which is then sealed by DNA ligase I/III.

Figure 6 provides a working model of how UV-DDB may act to stimulate MUTYH activity. Our previous work has suggested that UV-DDB arrives prior to OGG1 at sites of 8-oxoG at telomeres (25). Since many glycosylases are inhibited when their substrates are embedded in a nucleosome (39–47) future experiments will be necessary to determine whether UV-DDB is capable of stimulating MUTYH excision of 8-oxoG:A mispairs in the context of nucleosomes. No group has yet reported on whether MUTYH activity is inhibited when 8-oxoG:A is placed in a nucleosome. Data presented in this study shows that under optimal conditions UV-DDB can stimulate MUTYH excision of A from an 8-oxoG:A pair 4–5-fold. Furthermore, EMSA bulk studies showed that UV-DDB can displace MUTYH from abasic sites and forms transient complexes. Single molecule techniques combined with a DNA tightrope assay directly showed that UV-DDB was capable of displacing Qdot-labeled MUTYH from abasic sites. Finally, dual color Qdot experiments with UV-DDB and MUTYH indicated transient interactions in which MUTYH showed increased motility and dissociation from DNA. These data provide strong support for a direct role of UV-DDB in helping to turn over MUTYH from its product, abasic sites. Previous work from our laboratory indicated that UV-DDB can stimulate APE1 8-fold on abasic sites and stimulate Pol β incorporation some 30-fold (25). These data together with our present study, suggest that UV-DDB may play a direct role in the removal of A across from 8-oxoG in cells by long-patch BER (Figure 6). Future experiments using a reconstituted system of UV-DDB, MUTYH, APE1, PCNA, Pol λ and DNA ligase I/III as well as cell experiments will be necessary to provide more support for this model.

Defects in both MUTYH and DDB2 are implicated in carcinogenesis. MUTYH mutations are associated with increased colon cancer (10). The studies presented here have important implications in that DDB2 KO mice show increased rates of spontaneous tumor formation. In a chemical/irritant model of colon cancer, DDB2 KO animals showed more and larger tumors (23,24). Finally correlative studies suggest that high levels of DDB2 are protective in human colon cancers (48). Future studies in cell and animal systems will be necessary to understand the extent to which UV-DDB is directly involved in the removal of 8-oxoG:A mispairs and how loss of DDB2 may increase oxidant-induced tumors.

## DATA AVAILABILITY

All raw data is available from the senior author and can be released upon request, vanhoutenb@upmc.edu.

## Supplementary Material

gkab591_Supplemental_Files
