# Supplementary material for: Single molecule analysis indicates stimulation of MUTYH by UV-DDB through enzyme turnover
Source: Nucleic Acids Res. 2021 Jul 7;49(14):8177–88. doi: 10.1093/nar/gkab591 (PMC8373069; doi:10.1093/nar/gkab591)
Supplement: gkab591_Supplemental_Files [file gkab591_supplemental_files.zip › MUTYH_Supple_figure_0610921_FIN.pdf]

Single molecule analysis indicates stimulation of MUTYH by UV-DDB through enzyme turnover

Sunbok Jang*[1,2], Matthew A. Schaich*[1,2], Cindy Khuu[3], Brittani L. Schnable[1,4], Chandrima Majumdar[3], Simon C. Watkins[1,5], Sheila S. David[3], and Bennett Van Houten[1,2,4]

Supplementary Figures and legends

A

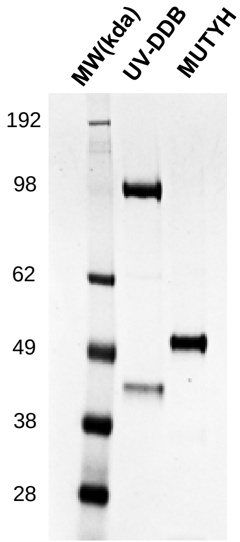

B

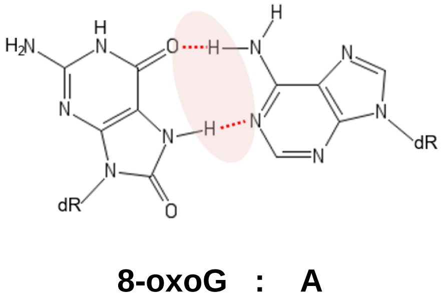

5'-CCG AGT CAT TCC TGC AGC GAZ TCC ATG GGA GTC AAA T-3'
3'-6FAM '-GGC TCA GTA AGG ACG TCG CTA AGG TAC CCT CAG TTT A-5'

C

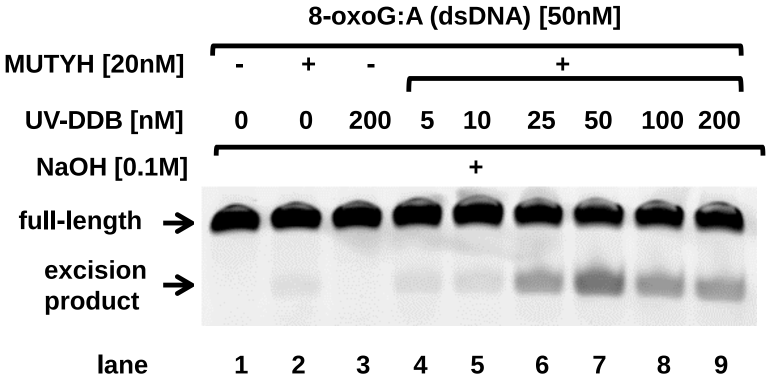

D

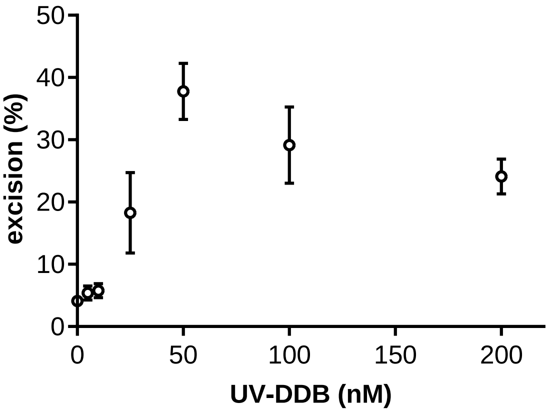

Figure S1. purified proteins, substrate chemical and MUTYH glycosylase activity in the absence or presence of UV-DDB, Related to Fig1.

(A) Coomassie stain of SDS-PAGE showing purified proteins used in this study. **(B)** Chemical structure of 8-oxoG:A and following oligonucleotide sequence (37bp dsDNA) was used in this study. (Z represents 8-oxoG) **(C)** Effect of UV-DDB concentration on stimulation of MUTYH excision. 8-oxoG:A was incubated with MUTYH and/or increasing amount of UV-DDB for 2.5hr at 37°C. The reaction was immediately stopped by adding 2X loading dye with 0.1M NaOH followed by heating 95°C for 5mins then quickly chilling on ice for 5mins. **(D)** Quantification of (C). Percent of total DNA that was excised by MUTYH plotted as a function of UV-DDB concentration. Data shown as the mean of three experiments ± s.d.

A

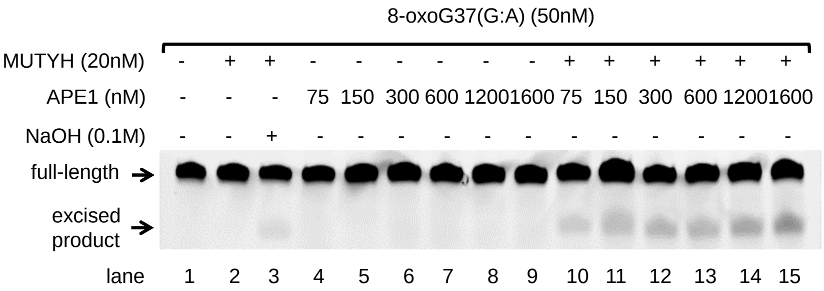

8-oxoG37(G:A) (50nM)
MUTYH (20nM) - + + - - - - - - + + + + + +
APE1 (nM) - - - 75 150 300 600 1200 1600 75 150 300 600 1200 1600
NaOH (0.1M) - - + - - - - - - - - - - - -
full-length
excised product
lane 1 2 3 4 5 6 7 8 9 10 11 12 13 14 15

B

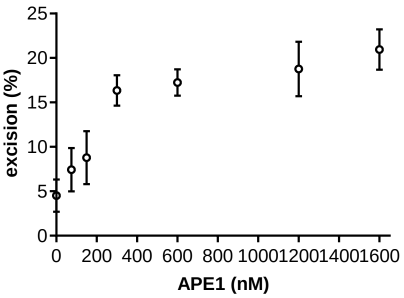

excision (%)
0
5
10
15
20
25
0 200 400 600 800 1000 1200 1400 1600
APE1 (nM)

C

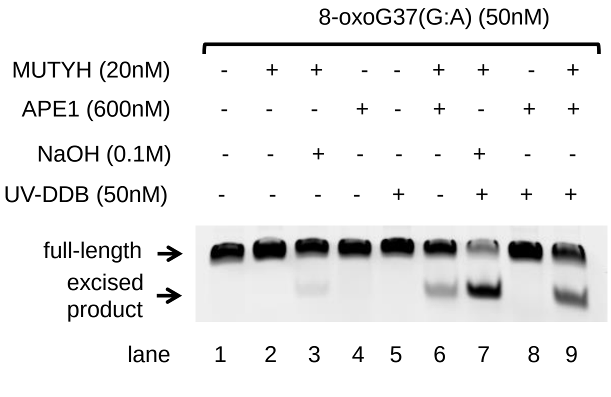

8-oxoG37(G:A) (50nM)
MUTYH (20nM) - + + - - + + - +
APE1 (600nM) - - - + - + - + +
NaOH (0.1M) - - + - - - + - -
UV-DDB (50nM) - - - - + - + + +
full-length
excised product
lane 1 2 3 4 5 6 7 8 9

D

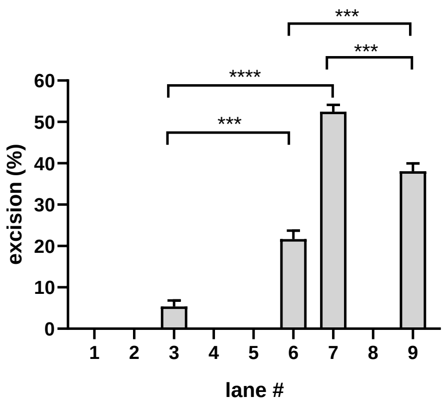

***
***
****
***
excision (%)
0
10
20
30
40
50
60
1 2 3 4 5 6 7 8 9
lane #

E

F

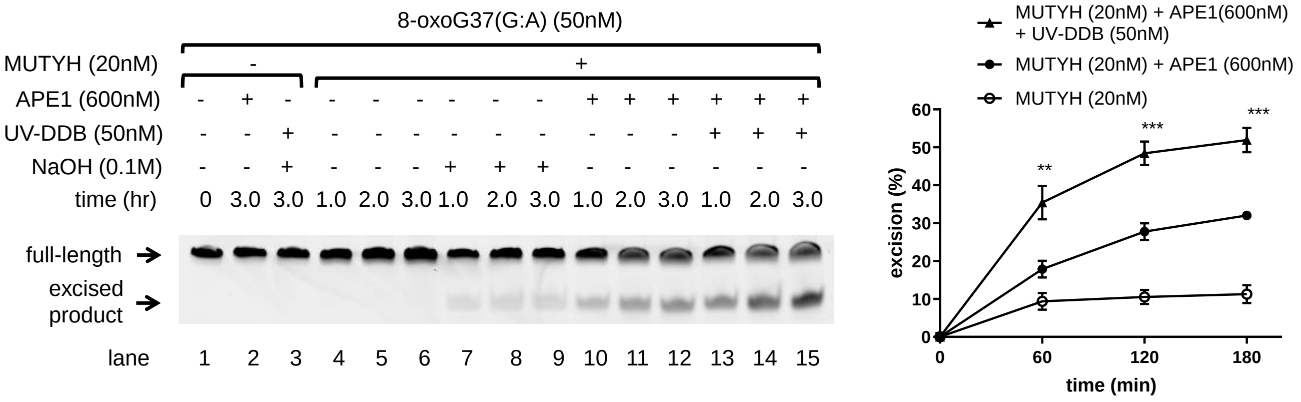

Figure S2. Effect of APE1 and UV-DDB on stimulation of MUTYH, Related to Fig1.

(**A**) Effect of APE1 concentration on stimulation of MUTYH excision. 8-oxo37 (G:A) was incubated with MUTYH and/or increasing amount of APE1 for 1.5hrs at 37°C. The reaction was immediately stopped by adding 2X loading dye followed by heating 95°C for 5mins then quickly chilling on ice for 5mins. **(B)** Quantification of (A). Percent of total DNA that was excised by MUTYH plotted as a function of UV-DDB concentration. Data shown as the mean of three experiments ± s.d. **(C)** Effect of APE1 or UV-DDB on stimulation on MUTYH excision. 8-oxoG37(G:A) was incubated with MUTYH only, MUTYH + APE1, MUTYH + UV-DDB or MUTYH + APE1 + UV-DDB for 2hrs at 37oC and separated by denaturing polyacrylamide electrophoresis. **(D)** Quantification of (C). Percent of total DNA that was excised by MUTYH plotted as lane number. Data shown as the mean of three experiments ± s.d. (*** P<0.001, **** P<0.0001)

(E) Effect of APE1 and UV-DDB on stimulation on MUTYH excision. 8-oxoG37(G:A) was incubated with MUYTH only, MUTYH + APE1, or MUTYH + APE1 + UV-DDB for 3hrs at 37°C and separated by denaturing polyacrylamide electrophoresis. **(F)** Quantification of (E). Percent of total DNA that was excised by MUTYH plotted as a function of time. Data shown as the mean of three experiments ± s.d. (** P<0.01, *** P<0.001)

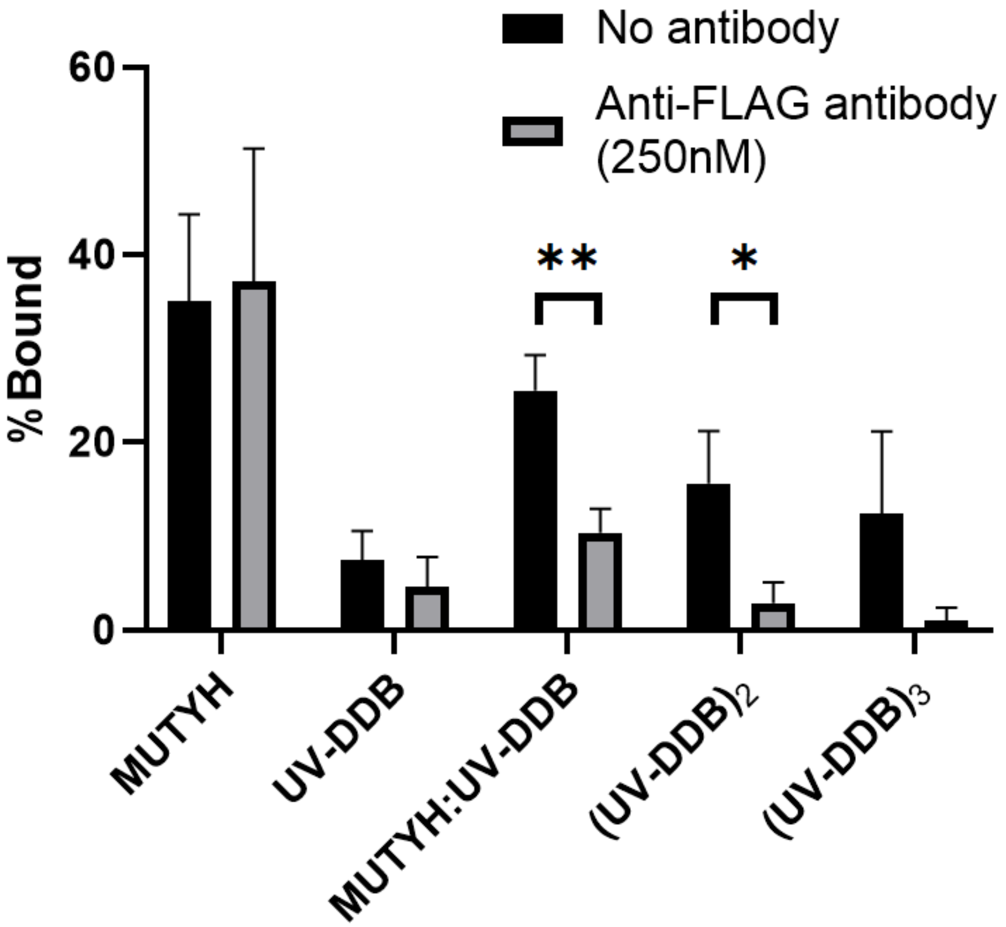
No antibody
Anti-FLAG antibody (250nM)
% Bound
60
40
20
0
**
*
MUTYH
UV-DDB
MUTYH:UV-DDB
(UV-DDB)2
(UV-DDB)3

Figure S3. Anti-FLAG antibody addition specifically binds complexes containing UV-DDB, Related to Fig 2.

Complexes containing FLAG-tagged UV-DDB are converted to supershift bands upon treatment with 250 nM anti-FLAG antibody, resulting in a reduction of intensity at the original band position. When a complex does not contain UV-DDB (such as the MUTYH band), no reduction is seen. Black bars represent the percentage bound of the complex prior to anti-FLAG antibody treatment, and gray bars represent the percentage bound of the complex after treatment. Data shown as the mean of two measurements from three experiments ± s.d. A single asterisk indicates a p-value of < 0.05 and two asterisks represent a p-value of <0.01.

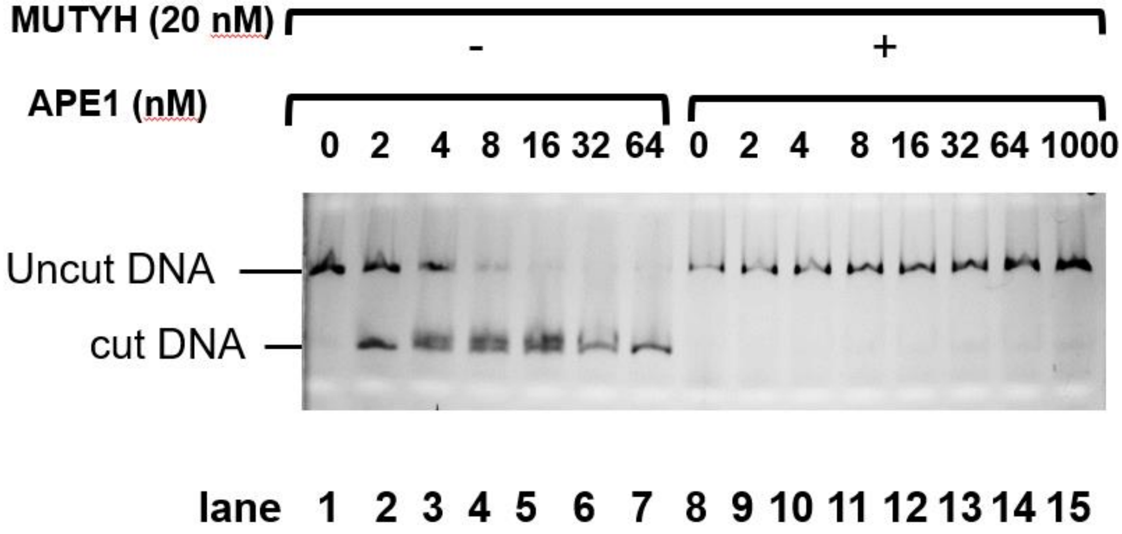

Figure S4. MUTYH product affinity prevents nicking by APE1, Related to Fig 2.

After performing the binding reactions in Fig. 2, the samples were immediately quenched in F-dye and separated on 10% denaturing PAGE. Without MUTYH present, APE1 uses its endonuclease activity to cleave the substrate at the abasic site (lanes 1-7). When MUTYH is added (lanes 8-15), the DNA substrate is not significantly cleaved by APE1, even at 1 uM concentration. Data shown was a representative image from 3 independent experiments.

A

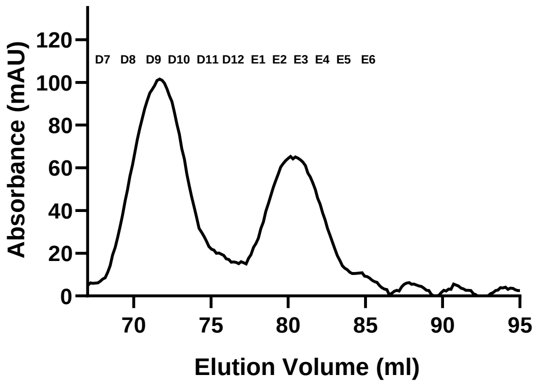

B

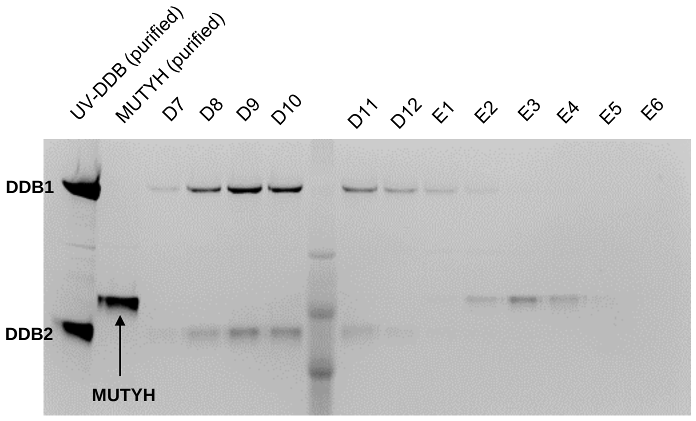

Figure S5. Size exclusion column chromatography, Related to Fig3.

(A) Mixtures of MUTYH and UV-DDB were subjected to analytical size exclusion column chromatography. Elution volume and faction number with the UV280 absorbance are indicated. **(B)** Fractions indicated (A) were analyzed by SDS-PAGE and silver-staining.

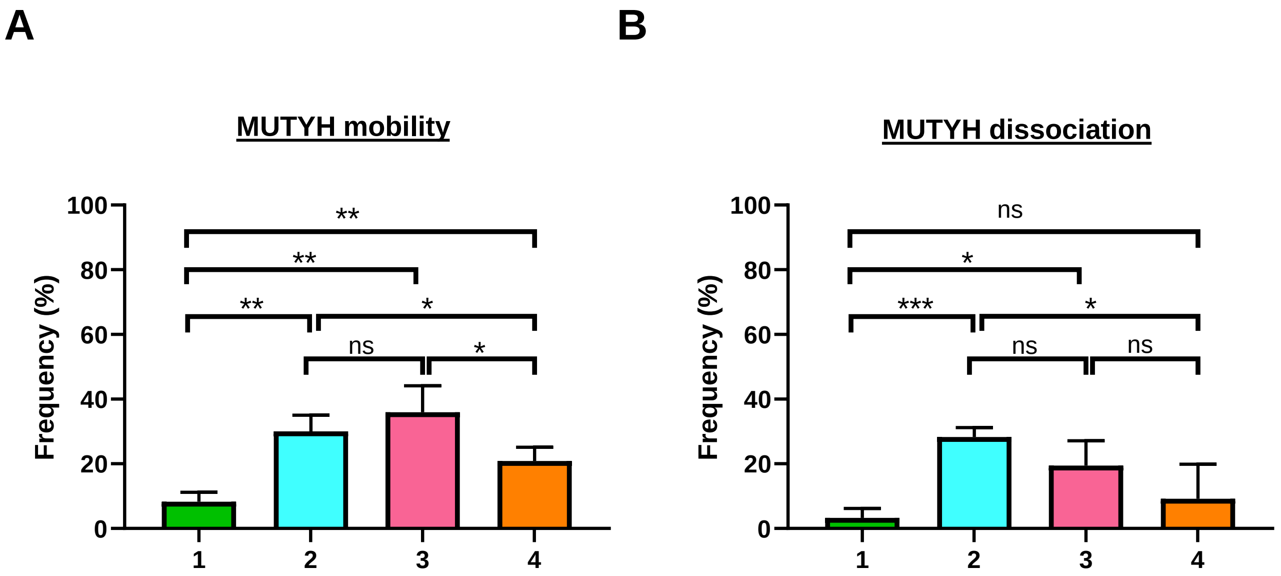

1. **MUTYH-mHis-605Qdot**
2. **MUTYH-mHis-605Qdot**
   in the presence of 1x unlabeled UV-DDB
3. **MUTYH-mHis-605Qdot**
   in the presence of UV-DDB-gFlagb-705SAQdot (not co-localized)
4. **MUTYH-mHis-605Qdot**
   in the presence of UV-DDB-gFlagb-705SAQdot (co-localized)

Figure S6. Single molecule analysis reveals UV-DDB stimulates turnover of MUTYH by facilitated mobility and dissociation. Related to Fig3 & 4.

(A) Mobility of 605Qdot-labeled MUTYH on DNA tightropes containing abasic sites (THF) in the absence (1) or presence (2,3 & 4) of UV-DDB. Bar graph data shown as means ± s.d with three (1,2) or four (3,4) independent experiments. (* $p<0.1$, ** $p<0.01$ by two-tailed Student's t test). **(B)** Dissociation of 605Qdot-labeled MUTYH on DNA tightropes containing abasic sites (THF) in the absence (1) or presence (2,3 & 4) of UV-DDB. Bar graph data shown as means ± s.d with three (1,2) or four (3,4) independent experiments. (* $p<0.1$, *** $p<0.001$ by two-tailed Student's t test)

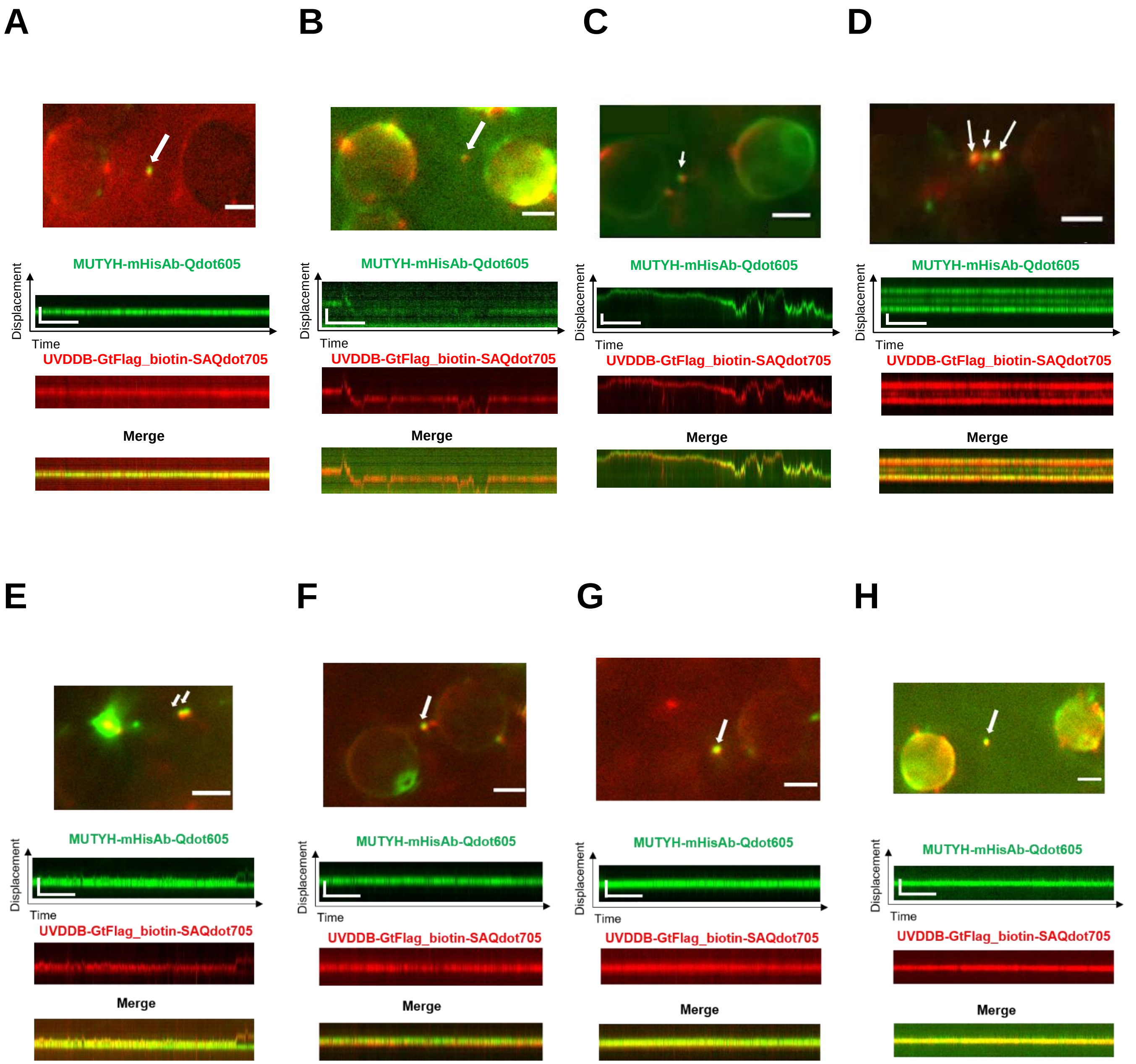

Figure S7. DNA tightrope assay showing co-localization of UV-DDB and MUTYH, Related to Fig3.

(A-H) Additional still frames and corresponding kymographs of co-localized MUTYH and UV-DDB (MUTYH: green, UV-DDB: red, and merge: yellow). Top, scale bar represents 2.5 μm; arrows point to co-localized particles. Bottom, horizontal and vertical scale bars represent 50s and 2kb, respectively.

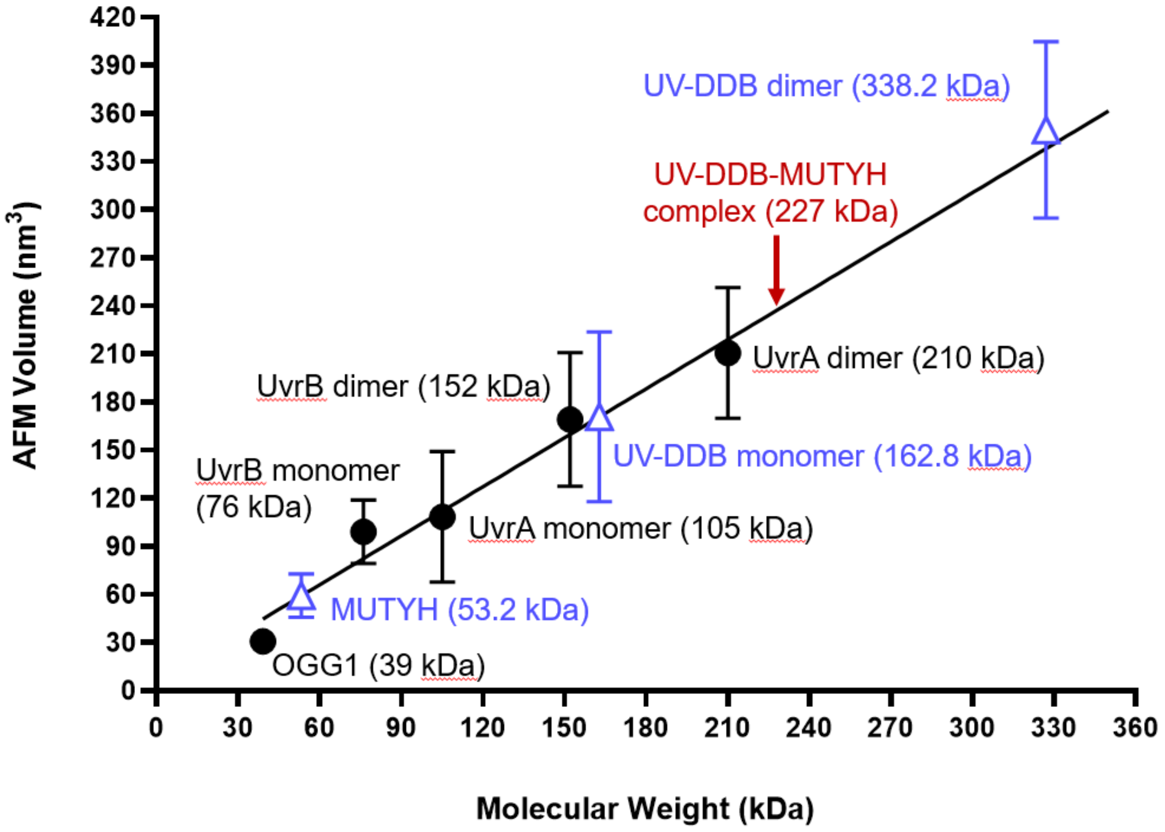

Supplementary Figure 8. The standard curve used to determine molecular weight of complexes, related to Fig 5. Solid black circles are proteins of known molecule weight (*n* between 600 and 1000) plotted against volume determined by PeakForce Tapping AFM. The linear regression (solid black line) is AFM Volume (nm^3) = 1.019 x Molecular Weight (kDa) + 5. The molecular weights for free MUTYH (*n* = 491), UV-DDB and UV-DDB dimer (*n* = 470) are indicated by purple triangles. The red arrow indicates the expected molecular weight for the MUTYH-UV-DDB complex.

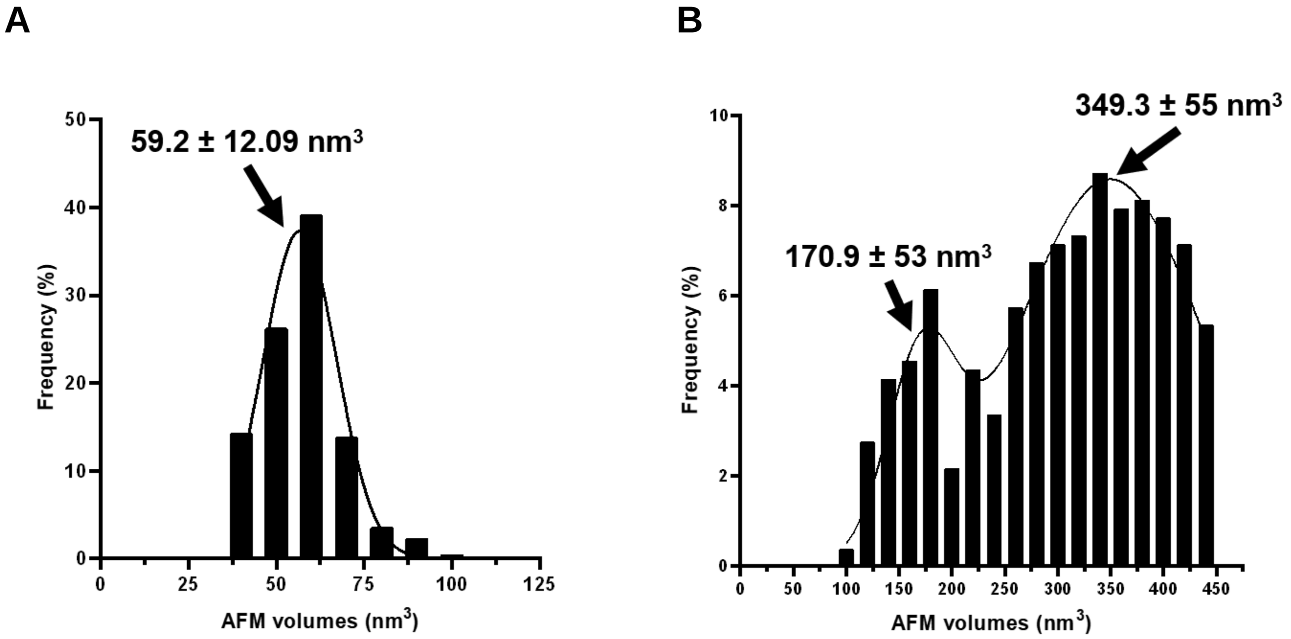

Supplementary Figure 9. The histogram of MUTYH and UV-DDB AFM volumes, related to Fig 5. (A) The histogram and Gaussian fit of AFM volumes of free MUTYH (*n* = 491) imaged by AFM. Gaussian is labeled with mean and standard deviation (59.2 ± 12.09 $nm^{3)}$. **(B)** The histogram and Gaussian fit of molecular weights of free UV-DDB (*n* = 504). Gaussian is labeled with mean and standard deviation for UV-DDB monomer (170.9 ± 53 nm^3) and UV-DDB dimer (349.3 ± 55 nm^3).

Descriptions of Videos

Video 1: 605 nm-Qdot labelled-MUTYH induced motion on a DNA tightrope containing abasic sites in the presence of unlabeled UV-DDB corresponding to Figure 3F. The data is collected at 11.38fps and are played back at 30fps.

Video 2: 605 nm-Qdot labelled-MUTYH induced motion and dissociation from a DNA tightrope containing abasic sites induced by unlabeled UV-DDB corresponding to Figure 3H. The data is collected at 11.38fps and are played back at 30fps.

Video 3: Co-localization of 605 nm-Qdot labelled-MUTYH with 705 nm-Qdot labelled-UV-DDB. Middle complex of both proteins undergoes constrained motion on a DNA tightrope containing abasic sites into a non-motile MUTHY-UV-DDB complex (on right), and does not slide past this stationary complex corresponding to Figure 4C. The data is collected at 1.74fps and are played back at 12fps.
